# Switching between tolerance and immunity: Do counter‐acting gene networks dictate Langerhans cell function in the skin?

**DOI:** 10.1002/bies.202100072

**Published:** 2021-03-30

**Authors:** Clare L. Bennett

**Affiliations:** ^1^ Department of Haematology University College London (UCL) Cancer Institute London UK

Immune responses must be carefully regulated at barrier sites such as the skin to ensure that we respond to infection, but not the daily innocuous insults that may enter the body. Langerhans cells (LC) are a unique population of antigen presenting cells that form a contiguous network throughout the outer epidermal layer of the skin.^[^ ^1^ ^]^ However, despite their discovery in the 19th century, and the remarkable conservation of LC from zebrafish to mice and men, our understanding of the role LC play in the balance of skin immunity remains opaque. This ambiguity stems partly from the fact that LC can migrate out of the epidermis to draining lymph nodes (LN) to prime naïve T cells.^[^ ^2^ ^]^ But dendritic cells (DC), the professional antigen presenting cells of the dermis, are sufficient to activate T cell responses to most cutaneous infections. These and other findings have led to the emergence of the dominant concept in recent years that LC may be more important for regulating T cell responses to harmless antigens in the skin. In this case, the major outstanding question challenging the field is: what is it about LC biology compared to dermal DC populations that favors the induction of T cell tolerance?

In this issue of BioEssays, Polak and Singh have sought to provide insight into this question by considering the unique tethering of LC within epidermal keratinocytes, and the signals derived from the cellular attachments formed therein.^[^ ^3^ ^]^ Bringing a unique perspective from their previous work using genomic and epigenetic analysis of primary human LC,^[^ ^4^ ^]^ they hypothesize that the existence of LC in “tolerogenic” or “immunogenic” states is determined by counter‐acting gene regulatory modules. These are linked to the disruption of cell contacts in the epidermis via a shared core activation/maturation gene module.^[^ ^3^ ^]^ This hypothesis is centered on the proposed pivotal roles played by interferon regulatory factors (IRF), previously shown by the authors and others to play key roles in the T cell‐stimulatory properties of DC and LC. Thus, they posit that in healthy skin loss of contact with the epidermis leads to upregulation of IRF4, which acts as a master switch to maintain expression of a tolerogenic gene program. They further hypothesize that inflammation and infection leads to the TNFα‐dependent activation of the related factor, IRF1, which counter‐acts the IRF4‐dependent gene program and takes over control of LC, which are now proficient at initiating T cell immunity upon arrival in the LN. The exciting nature of this hypothesis lies in the concept that competing gene regulatory networks interact with residency signals from the epidermis to control LC function, and provides a compelling argument to test with future experiments. By necessity, much of the supporting data for Polak and Singh's model depends on analysis of LC that have migrated out of skin cultures in the presence or absence of TNFα, but it remains to be seen how these cells compare to LC arriving in draining LN in vivo, and how signaling pathways from cell‐adhesion molecules and TGFβ intersect with IRF4 and IRF1 in these cells. Use of genetically modified murine models, and analysis of patient LN would help to validate these findings and provide a more mechanistic understanding.

Within the field, open questions still remain about the relative importance of the proposed homeostatic role for LC in the skin. The term ‘‘tolerance’’ is a broad concept and future studies are needed to shed light on the functional impact of IRF4‐ versus IRF1‐programmed LC in LN; how do they interact with conventional and/or regulatory T cells, and how do these interactions control potentially auto‐reactive T cells? Furthermore, this hypothesis assumes a dominant role for LC at the point of T cell priming in LN, but we and others have shown that interaction between LC and T cells in situ within the epidermis may also control T cell function.^[^ ^5^ ^]^ The novel approaches and hypotheses such as those used by Polak and Singh will provide insight towards addressing these questions and begin to reveal the importance of these unique and enigmatic cells in the skin.

## CONFLICT OF INTEREST

The author declares no conflict of interest.
